# “Whenever I help her, I am also expecting her vagina in return”: a qualitative analysis to explore men’s and adolescent girls’ perceptions of the impact of the COVID-19 pandemic on the sexual behaviour and health of adolescent girls in rural western Kenya

**DOI:** 10.1136/bmjph-2024-001214

**Published:** 2024-11-14

**Authors:** Enid Awiti, Sophie Young, Garazi Zulaika, Fredrick Odhiambo Otieno, Elizabeth Nyothach, Penelope A Phillips-Howard, Supriya D Mehta, Linda Mason

**Affiliations:** 1 Nyanza Reproductive Health Society, Kisumu, Kenya; 2 University of Illinois, Chicago School of Public Health, Chicago, Illinois, USA; 3 Clinical Sciences, Liverpool School of Tropical Medicine, Liverpool, UK; 4 CGHR, KEMRI, Kisumu, Kenya

**Keywords:** COVID-19, public health, female

## Abstract

**ABSTRACT:**

**Introduction:**

The COVID-19 pandemic caused school closures, which intensified the negative sexual and reproductive health (SRH) of adolescent girls in sub-Saharan Africa (SSA), including increases in transactional sexual partnerships, gender-based violence, risk of early pregnancy and sexually transmitted infections (STIs). We conducted a qualitative study to understand how adolescent girls experienced and reacted to the pandemic restrictions and perceived consequences on their schooling and sexual behaviours. In parallel, we sought community men’s perceptions and opinions on the same issues.

**Methods:**

Set in rural western Kenya, the study used six focus group discussions with adolescent girls and five with community males aged 19–41 years, conducted from June 2022 to January 2023.

**Results:**

Thematic analysis identified three key themes, parallel in girls and men: (1) *impacts of COVID-19 on schooling*: girls reported uncertainty around ever returning to education, consequently losing motivation to study, which was also observed by men; (2) *drivers that increased sexual activity*: transactional sex became a greater necessity due to acute poverty, while opportunity escalated through additional leisure time and (3) *sexual behaviours and practices*: girls increased the number of partners and frequency of sexual encounters, with power-imbalances reported. Men believed they were assisting girls through transactional sex but this was often conditional on receiving sex in return, viewing themselves as victims of girls’ seductive advances, and blaming girls for transmitting STIs.

**Conclusion:**

School closure jeopardised girls’ SRH through acute poverty and increased opportunity for sexual exposure. Mitigation methods are needed now to prevent girls bearing the brunt of ensuing societal disruption and acute poverty in future catastrophes. Deeper understanding of men’s attitudes and behaviours towards adolescent girls are needed to improve the foundation for working with them to reduce power imbalance and compulsion in sexual interactions with adolescent girls.

**WHAT IS KNOWN ON THIS TOPIC**

**WHAT THE STUDY ADDS**

- This study provides a nuanced understanding of the factors underlying COVID-19-related increases in pregnancies and STIs for adolescent girls.
- Girls ceased to study due to loss of motivation arising from the uncertainty of ever returning to school.
- Increased poverty was perceived to exacerbate transactional sex among adolescent girls, a greater number of partners and frequency of sexual activity along with heightening power imbalance.
- Men reported they wanted to assist girls, but generally expected sex in return, seeing themselves as victims of schoolgirls’ advances and blaming them for spreading STIs.

**HOW MIGHT THE STUDY AFFECT RESEARCH, PRACTICE, POLICY**

- These data highlight the need to provide safeguarding mechanisms to protect girls from future catastrophic events that ultimately affect their schooling and sexual and reproductive health via pathways of poverty and increased exposure to sexual vulnerability.
- This research recognises the role of men’s attitudes and behaviours towards vulnerable school-aged girls, and notes the necessity of engaging with them in changing behaviours that consequently harm the health and wellbeing of adolescent girls.

## Introduction

 The COVID-19 pandemic caused school closures in over 190 countries and affected approximately 1.6 billion students worldwide.^1^ Globally, school closures averaged 41 weeks with considerable negative impacts on learning and is projected to have significant long-term consequences for education outcomes, future employment and household incomes.^1^,^3^ Additionally, concern has been raised on the loss of the protective effect of schooling on gender inequalities, and sexual and reproductive harms.^4^ A comprehensive study of multiple countries conducted by the World Bank demonstrates that staying in school shields girls from early marriage, teen pregnancy and other sexual and reproductive health (SRH) harms, including HIV and sexually transmitted infections (STIs).^5^

Emerging evidence indicates that school closures intensified the negative SRH and schooling outcomes of vulnerable adolescent girls.^6^ Previous progress in reducing adolescent pregnancy and child marriage stalled during the pandemic, with one systematic review^7^ concluding that containment measures put in place likely contributed to high rates of teen pregnancies, due in part to the reduction in access to SRH preventive services, including for contraceptives and HIV and STI prevention and treatment*.*^8 9^ Limiting or omitting SRH services for adolescent, and/or reducing information about their availability resulted in higher rates of unwanted pregnancy, early marriage, unsafe abortion, pregnancy-related deaths, STIs and reproductive tract infections, and lower rates of HIV treatment.^10^

In Kenya, the COVID-19 outbreak closed schools in March 2020; only secondary students due to take their final year National Exams were allowed to return in October for preparation, with schools fully reopening in January 2021.^8 11^ Emerging evidence among Kenyan adolescent girls and young women (AGYW) report an increased reliance on transactional partnerships, an increase in gender-based violence and greater risk of early pregnancy and deprivation of partner emotional support during the COVID-19 restrictions.^12^ In western Kenya, the current study setting, school closures were associated with increased school dropout and sexual activity and twice the risk of becoming pregnant.^13^

Few studies have investigated perceptions of girls and compared these with views of males. The present qualitative study was designed to better understand how AGYW experienced life as schoolgirls during the COVID-19 pandemic. To date, while quantitative studies have documented behaviour and outcomes, we have little understanding of the drivers behind these. We also sought the opinions of men from the same communities in which the girls lived, as they are highly influential, seen as the main wage earners and heads of household in this patriarchal society, and may have been instrumental as leaders during the COVID-19 pandemic. They are also (potential) sexual partners of AGYW, thus their opinions and perceptions may stem from varying roles. We undertook this comparative analysis of AGYW and community men’s perspectives on the impact of COVID-19-related school closures on schoolgirls to understand how adverse impacts may be anticipated and prevented in future crises.

## Methods

### Study area and population

The study was conducted in Rarieda Subcounty in Siaya County, rural western Kenya. With a population of 152, 570,^14^ Siaya residents are primarily of the Luo ethnic group and are predominantly subsistence farmers, fisherfolk and gold miners.^15^ High rates of HIV and STIs are reported among AGYW.^16^ Additionally, there are elevated rates of school drop-out in the region, with only 8% of women completing secondary school.^16^

### Recruitment

Participants were recruited from the Cups and Community Health study (CaCHe), a substudy of the Cups or Cash for Girls (CCG) Trial.^17^ CaCHe evaluated the effects of menstrual cups on the vaginal microbiome, bacterial vaginosis (BV) and STIs in 436 AGYW aged 14–25 years at enrolment.^18^ During a follow-up survey visit (April through June 2022), after the schools had re-opened, AGYW were asked if they were willing to participate in qualitative focus group discussions (FGDs). From those responding positively, an enumeration list was created with stratified simple random sampling (to ensure representation across the study area) and participants selected until sufficient group sizes were reached. Men from the community were recruited opportunistically during field visits by study staff, with snowballing used to recruit additional participants.^19^ Eligibility included coming from the same study area and consent to study participation. Given the predominant jobs locally, we targeted motorcycle taxi drivers, fisherfolk, miners and farmers. Recruitment was not targeted to men based on a specific age range or relationship with an AGYW, and was meant to be generally inclusive of men from the same communities who may hold different roles in relation to girls (eg, parental or other authority figures, sexual partners, romantic partner, friends, neighbours, etc).

### Focus group discussion approach

Two semi-structured FGD guides were developed for AGYW and males, with overlapping themes enabling comparisons. The guides focused on participants’ perceptions, feelings and behaviours about COVID-19 school closures, and their sexual relationships and SRH (online supplemental file 1).

We aimed to conduct at least eight FGDs in total: four with AGYW and four with community males. Two additional FGDs were carried out with AGYW and one with men to ensure that no new themes were emerging and all conceptual categories in the guides were covered. Aiming for 10 participants per FGD, we invited 12 to each session to allow for non-attendance.

### Procedure

FGDs were conducted from June 2022 to January 2023. Eligible individuals who remained interested after the study was explained were invited to a scheduled FGD. Each participant was assigned a number to protect confidentiality (denoted in the quotes, eg, Girl 3). FGDs were conducted in a private room at a central location (eg, church or community hall) and lasted approximately 1.5–2 hours. FGDs were carried out in English, Swahili, Dholuo or a mixture, depending on participant preference. A Kenyan moderator (EA) led each discussion following the FGD guide, probing as necessary. All FGDs were audio recorded with participant permission. A male notetaker was used for discussions with men, and a female notetaker recorded FGD notes for the AGYW. The FGDs were transcribed verbatim and any Dholuo and/or Swahili was translated into English. The transcripts were then reviewed and compared against the original audio for accuracy by the moderator.

### Analysis

The transcripts were entered into NVIVO V.12 Pro Software. The analysis adopted both a deductive and inductive approach generating findings to meet our aims, while allowing participants’ views to emerge.^20^ We used thematic analysis to facilitate understanding of the participants’ behaviours and perspectives.^21^ The moderator (EA), joint first author (SY) and senior authors (SM and LM) read the transcripts for familiarity with the data. From this, general patterns emerged which were noted as initial themes. Detailed codes were assigned to these themes and built into the coding framework. The framework was edited dynamically until all transcripts were coded and a series of subthemes and themes identified. To reflect any overlap and redundancy, the framework and themes were amended iteratively, with further analysis undertaken to compare across and within AGYW and community men transcripts. Both first authors coded and analysed the data independently, and then compared results, discussing and resolving where any disagreement occurred. Direct quotes from the transcripts were selected for illustration.

### Patient and public involvement

Our team includes local Kenyan researchers who had key input into study planning, guide design and interpretation. No research participants were involved in this process. No patients were involved in the study. Prior to starting the CCG and CaCHe studies, we met with the community and undertook community engagement activities, getting advice from village chiefs, stakeholders, headteachers, parents, etc as well as informing them of our activities. We also work with the community, holding workshops to find appropriate ways to move forward with potential solutions and to disseminate our findings. We have found however that adolescent girls are somewhat reticent to contribute in these forums and we need to find ways to ensure active and full participation in the future.

## Results

A total of 11 FGDs were conducted with 107 participants: 6 with AGYW (n=54) and 5 with community males (n=53). All AGYW and 98% of men were of Luo ethnicity (one male participant was Luhya). Their demographic characteristics are summarised in table 1. For continuity within the text, we also refer to the AGYW participants as ‘schoolgirls’ or ‘girls’ to reflect their circumstances at the time recalled in the FGDs.

**Table 1:** Distribution of FGD participant characteristics

	AGYW, n=54		Community men, n=53	
**FGDcharacteristics**	**Number of participants**	**Duration**	**Number of participants**	**Duration**
FGD				
1	11	2 hours, 11 min	11	2 hours, 15 min
2	8	2 hours, 17 min	12	2 hours, 15 min
3	11	2 hours, 50 min	10	2 hours, 38 min
4	10	2 hours, 0 min	12	2 hours, 0 min
5	8	2 hours, 10 min	8	2 hours, 30 min
6	6	2 hours, 22 min		
**Participantcharacteristics**	N (%)		N (%)	
Mean age (range)	20.9 (18–24)		27.1 (19–41)	
Married	4 (7%)		25 (47%)	
Has children	13 (24%)		29 (55%)	
Currently in school	24 (44%)		1 (2%)	
Occupation	6 (11%)		53 (100%)	
	2 (4%) Farming		13 (58%) Motorcycle taxi	
	1 (2%) Hotel work		13 (25%) Fisherman	
	1 (2%) Salon		8 (15%) Miner	
	2 (4%) Shop attendant		2 (4%) Farmer	
			3 (6%) Businessman	
			1 (2%) Artisan	

AGYW had to report this as employment outside of home chores.

* Not mutually exclusive, some men had multiple jobs.

Table 2 presents the three key themes with associated subthemes that surfaced during discussions.

**Table 2:** Emerging themes and subthemes from focus group discussions

Theme	Subthemes	AGWY	Community men
Impacts of COVID-19 on schooling	Uncertainty in returning to educationLoss of motivation to study	YesYes	NoYes
Drivers thatincreased sexualactivity	PovertyOpportunity and leisure timePeer influence and pressure	YesYesYes	YesYesNo
Sexual behavioursand practices	Increased number and frequency of sexual partnersUsing or taking advantage of menPower imbalance	YesNoYes	YesYesYes

### Impacts of COVID-19 on schooling

#### Uncertainty in returning to education

Many girls spoke of uncertainty surrounding their educational futures and how the pandemic seemed to render their schooling vulnerable, especially for those who had been in their final year of secondary school. Some AGYW voiced fear that they would have to undergo a further year of study, others were certain they would have to drop-out without completing their education.

*We knewsometimes we are not going to complete school; we were going to stay at home and drop out of school.(Girl 5,FGD6)*

Men echoed this disruption to girls’ education and spoke of the uncertainty around future school requirements. They also voiced that girls were not attentive to their studies during school closures.

*Schooling might proceed to even five years. She doesn’t study or revise every day. After doing the house chores at home, the remaining part of the day is just walking around the village. (Male 5,FGD5)*

#### Loss of motivation to study

Most girls reported they studied diligently at the beginning of the school closures, but as time went on, they lost motivation due to the repeated closure extensions and uncertainty around school reopening. While a few girls stated they never studied during this period, many voiced that they found it difficult to concentrate at home.

*The reason why we left studying, you hear that next month school is opening on a certain date. On that date you hear that the disease has increased, Corona has been found somewhere. They again fix [a date] for next month, the next month again it is postponed. So, we said school has finished and we should stop bothering ourselves. (Girl 6,FGD3)*

*I studied for the first one to three weeks, [……………] and thereafter we stopped studying since the closure was extended for two more weeks until they continued with the extension until we lost hope.(Girl 3,FGD5)*

Just two girls conveyed feeling positive about school closures, believing that time off from school benefitted their education and provided time to prepare for exams and collect money for school fees.

### Drivers that increased sexual activity

#### Poverty

One main theme emerging from both girls’ and men’s narratives centred around the pandemic-related increase in poverty and economic insecurity many families experienced. Many participants, AGYW and men alike, believed this was a key driver of a perceived increase in sexual activity among AGYW. Some girls reported feeling obligated to sleep with men for money to provide for their families, others were compelled to do so to meet their own basic needs.

*Some are pushed into having sex in order to support their parents and that is sometimes due to poverty, so they decide to get into any form of job to support their parents.(Girl 4,FGD2)*

*Due to peer pressure, most girls were being forced to have sex for money. As in, their families did not have enough income so they couldn’t get most of their basic needs like clothing. (Girl 4, FGD3*)

Some girls suggested that engaging in transactional sex came from a sense of personal duty, and also from parental pressure. Several girls stated that parents pushed their daughters into sexual exchanges to supplement household income during the pandemic. Pressure arose particularly from mothers wanting their daughters to bring home food or small luxuries.

*Some parents could send their daughters to go have sex with some people in order to get money that could be used to buy food. (Girl 8,FGD4)*

Men also perceived an increase in transactional sex, and spoke of their role, acknowledging that often the main reason girls turned to them was for financial support.

*The girl child during Corona, they depended on the motorbike riders. The reason why I say that they depended on motorbike riders, financial earnings for the people went down now the motorbike riders were the only people who could have money, now they loved us. Now sometimes she could want to buy her things, it would force them to be friends with us to get the money.(Male 5,FGD3)*

Men’s opinions about transactional sex with AGYW could be contradictory. Acknowledging girls’ financial needs as a motive for sex, many of them felt as though they were helping these girls and used the words ‘*help*’ or ‘*assist*’ repeatedly as per: “*Yes, if I could get many beautiful ones I could just help” (Male 3, FGD4*). However, men also indicated this ‘*help*’ came at a price and that the girls ‘*owed*’ them for that help, particularly if financial assistance was repeated.

*There’s no way a woman suffering can ask me for help and I help her for free. Let us be honest, in this situation there’s no way you can tell me that if a girl comes and ask for help, that I help her for free.(Male 8,FGD1)*

*For example myself, I am helping a girl and providing everything she needs and whenever I help her, I am also expecting her vagina in return. Do you understand my point? (laughter) and so I also expect her to assist me whenever I need her help. (Male 2,FGD4)*

Men’s own motives for ‘helping’ girls were rarely discussed and that a price had to be paid for help given was not questioned. When probed, one man stated ‘*As for that, the hyaena must feast on the sheep’ (Male 2, FGD4*).

Among some men who spoke of the girls’ financial hardships and need for assistance, some voiced resentment that they were targeted for their earnings. A few stated that girls used them for their money.

*The girls relied on us as their parents owing to the fact that some parents lost their jobs. We were still generating income so they could depend on us as fishermen. You find that most of the time they are after us just because of money.(Male 10,FGD2)*

And while this awareness of girls’ financial vulnerabilities was echoed by many men, only one man described the girl as the ‘*victim’*. His atypical narrative follows:

*During the pandemic, there was little or no money to acquire the daily bread, those who were working more so fishermen had money and the girls also knew this very well. Some of these men could assist the girls since they knew the girls could not reject money. These girls fall victims without knowing that these men are baiting them with money. These girls also had difficulties in accessing dignity packs and even food so they believe that befriending these guys will earn them something to take care of their need. (Male 8,FGD2).*

#### Opportunity and increased free time

A second identified driver of heightened sexual activity was increased leisure time. Girls frequently indicated that they spent their free time visiting their boyfriends and having sex.

*Because there is nothing to do, because even helping parents you can’t help parents from morning to evening, you will clean utensils and parents also won’t stay with you in the compound. You will get that time they have left; you also leave. So, that freeness was what was bringing the issue to do with sex to be high.(Girl 8,FGD1)*

Furthermore, the evening-to-morning curfews during the pandemic gave girls an opportunity to sleep away from home as travelling to and from their homes became more difficult, consequently some stayed with boyfriends overnight:

*Suppose you go visiting your boyfriend and so the curfew finds you there, it would force you to spend the night there and you leave early the next day.(Girl 1,FGD5)*

Men similarly reported that opportunities to interact with girls increased during school closures, with one boda boda driver reporting:

*They only revised after doing the house chores and could the rest of the day be wandering around. If she finds me with my motorbike, I will carry her and, in the process, end up at my place, and it was something that could happen daily for one week, yes, there was that time and the chances of becoming pregnant were also seriously high.(Male9,FGD4)*

#### Peer influence and pressure

Frequently girls reported that they faced increased pressure to have sex from their partners and peers during the pandemic. Girls relayed how they felt unable to refuse their partner’s advances and that pressure to copy their peers, including to have material gains, drove girls to engage in sexual behaviours.

*Due to peer pressure, most girls were being forced to have sex for money. As in, their families did not have enough income so they couldn’t get most of their basic needs like clothing. You go out there and find your fellow girl has nice clothes and want to be like her. Another thing is negative advice, you get advised that you are still young and energetic and beautiful. Why don’t you go outside there and get money instead of sitting here depending on the parents? The pressure and bad advice can also influence you because you may not have work at home, and at least I can make some money for myself to get everything I want.(Girl 4,FGD3)*

Girls also voiced they felt threatened by the influx of girls returning to villages and towns due to the pandemic, driving them to engage in sex with their boyfriends to keep them interested. However, this dynamic in relationships was not discussed in the sessions with men.

*My boyfriend found that there were many girls. The way he used to beg for your attention was reduced because there were so many options.(Girl 5,FGD5)*

### Sexual behaviour and practices

#### Increase in number and frequency of sexual partners

Some girls reported requiring more sexual partners during the pandemic to obtain monies to meet basic needs, while others reported more partnerships because of increased opportunity for pleasure. Regardless of reason, AGYW and men generally agreed that girls’ sexual activity increased, with more frequent encounters and more partners.

*Covid has already affected our families, such as simple basic commodities like lotion, pads among other things. So, they had to have more partners to enable them to cater for these personal needs.(Girl 6,FGD2)*

*Moderator: Did your friends tell you that they had more or less boyfriends?*

*P: More.*

*Moderator: More boyfriends, why?*

*P: If one does not have money, they find another one so she becomes a player.(Girl 4,FGD1)*

Most girls reported that they preferred to date older men (ie, ‘sponsors’) with greater financial resources. Men also agreed that girls preferred older and more wealthy men.

*Most girls were engaged with older people who were above their age because they’re the ones who had money unlike the younger ones who were hustlers. So, only the older guys were the ones who could manage taking them.(Male 3,FGD1)*

Just two girls reported that they had fewer partners due to the restrictions, opting to stay home and abide by the curfew due to fears of contracting COVID-19.

#### Using or taking advantage of men

Although mostly realising that girls often had sex out of financial necessity, men described how some girls were seductive and intentional with their behaviour and took advantage of them. Some spoke of being rendered ‘helpless’ by the girls. Others suggested that just being around schoolgirls presented a temptation, as reflected in the following quotes from boda boda drivers:

*You are the one riding and she has held you very tight, and you have feelings. You know once you have feelings and human beings are like machines, you will be absent minded, you may be absent minded until you get to a point that you were not to get to. Even if she is a schoolgirl, the style that she comes to you, or even falls on you. Sometimes it was like seven pm and you find her walking on the road, you find things get bad and you do what you were not to do.(Male 2,FGD3)*

*They also take advantage; she knows there are things that she needs, and the parents can’t provide for her what she needs. She takes the advantage after she… like number two has said. Style that you carry her how she will react on your back because you also know that she is a schoolgirl. If you carry her, you also convince her it starts there. You will realize that she is a schoolgirl once things have happened.(Male 7,FGD3)*

*There is this trait that God created us men with, whenever you see a beautiful girl, you just have to pursue her against all the odds with all your resources to win her.(Male 11,FGD4)*

Despite the awareness that girls engaged with them due to economic need, men still placed the responsibility for sexual encounters on the girls. This was at odds with their descriptions of schoolgirls as preferred sexual partners. Across all men’s FGDs, consensus was that men preferred sex with schoolgirls because they were cheaper to maintain, faithful, had fewer needs and were sexually inexperienced or virgins.

*They won’t ask for a guest room after seducing her, you will just finish your urge whenever you want it. But those with their houses if you go there you have to leave behind a bar of soap for cleaning the bed sheets, you must also bring her food, you cannot have intimacy without feeding her.(Male 3,FGD3)*

*If I say young, young are those who are just coming up, those who are clean, those whose minds are still not that developed well. School girls. Because you know that those whose minds are developed you may go broke.(Male 3,FGD3)*

*Those in school are sweeter compared to others, I don’t understand what happens with human beings, the forbidden tastes sweetest.(Male 11,FGD4)*

#### Power imbalance

Power dynamics left girls frequently reporting that they felt unable to request their partners to use condoms. Girls in a sexual relationship with older men also reported being unable to negotiate condom use:

*If you are dating an elderly person who provides for all your needs, you can’t have the courage to sit him down and discuss with him if you use a condom when getting intimate, especially when one is not her safe days. There is always that fear because he is the one in control.(Girl 3,FGD2)*

The men echoed this sentiment, suggesting that they held power in a sexual relationships. Deceptive practices like stealthing were also mentioned in a few instances, indicating that they believe they have the right to remove condoms without informing their partner.

*In the situation, when people are having sex, the life of the lady is in the hands of the man, he is the one who determines what to do because mostly the way I know it most men like it without the protection. She will try to observe you putting it on, but to some extent, you remove it and carry it in your hands, she won’t know, so the life of the lady is in the hands of the person she is having sex with. (Male 5,FGD1)*

While men generally voiced dislike of condoms, some men spoke of condoms as important for girls because generally girls did not want to become pregnant until completing their education. Men also felt it was necessary to prevent girls from infecting them with STIs. However, there was no mention of condoms as a means to prevent men from infecting girls.

*First, we don’t know who these girls are. You cannot sleep with a girl without a condom. You should put on three at once because some of these girls have syphilis.(Male 10,FGD4)*

*I prefer we use condom. It is not good to have unprotected sex with someone other than your wife because they may be sick. (Male 2,FGD3)*

## Discussion

Our study combines AGYW and men’s narratives regarding the impact of COVID-19 school closures on the lives and sexual relationships of schoolgirls. This approach aims to understand the perceived drivers and consequences for girls’ SRH and schooling, incorporating reflections gathered after schools reopened.^6 7 13 22^ All AGYW participants were in school prior to lockdown, with just 44% remaining as students at the time of data collection. We found similarities in girls’ and men’s narratives, namely higher rates of sexual activity stemming from increased poverty and increased leisure time. However, differences between girls’ and men’s perceptions of past behaviours and motivating factors, and among men’s own individual beliefs were discordant at times. These findings are considered below.

Girls’ and men’s narratives suggest two key areas in girls’ lives that were affected by the COVID-19 closures and likely to have long-term consequences. The first involves how the interruption in schooling affected girls’ education. During these closures, self-study was girls’ only option, although motivation was hampered by repeated delays in reopening and fears schooling would end. In these impoverished settings, fees and associated costs of schooling make repeating a year a non-viable option and leaving school without a certificate can impede future employment. In the Kenya 2022 Demographic and Health Survey,^23^ women’s past year employment rate was lowest for the 15–19 (13%) and 20–24 (41%) age groups. Notably, the employment rate was strongly correlated with education, at 65% for those with more than secondary education compared with just 45% for those with only secondary education, highlighting the lifelong impact of disrupted schooling. In anticipation of future pandemics, solutions for alternative learning and motivational strategies need to be prioritised in low-and-middle-income country (LMIC) settings to ensure that girls are not disadvantaged economically for life. Alternative strategies that address the hurdles of remote learning in low-resource settings may include government initiatives or public-private partnerships that employ a variety of strategies: developing and hosting high-quality learning across modalities (such as internet, television and radio), providing teacher training for remote learning, improving remote learning infrastructure and technology (eg, supplying internet-connected devices, internet data quotas, in-person visits to areas with poor internet signals and paper take-home packets) and making efforts to rekindle students’ interest and participation in learning.^24 25^

The second implication from our findings was that despite curfews, restrictions and extended school closures, schoolgirls faced higher SRH risks than prepandemic. This agrees with our quantitative data that showed higher rates of pregnancy, BV and STIs during the COVID-19 period compared with the pre-COVID-19 period.^13 26^ FGD participants described two causal pathways: (1) the loss of structure in girls’ lives and spare time to pursue sexual relationships and (2) resiliency behaviours in response to an increase in poverty, which drove higher rates of transactional sex. While normative in the study area,^27 2^,^8^ participants, both male and female, were of the opinion that transactional sex was markedly higher during the pandemic as pressures to obtain money or items mounted, and ‘exposure’ due to additional leisure time in the community was greater. Our participants’ narratives provide a rational explanation for the increase in pregnancy, abortion and STIs reported in other studies in LMIC during the pandemic.^13 26 29 30^ The economic instability caused by the pandemic had gendered impacts with girls becoming more of a commodity, and their bodies were used as a service to exchange for money, food and other necessities. This paper further highlights the excessive risks faced by girls during school closures, and illustrate the need for communities and government agencies to implement interventions that mitigate and minimise such risks.

Participants noted heightened power imbalances in transactional relationships. In our study, men—and a few girls—primarily described choosing not to wear condoms because they did not like them. Most girls, however, expressed that they lacked agency to request that their partner wear a condom. Those who do make such requests may then be subjected to male stealthing. Although girls’ lack of agency is not unusual in transactional relationships,^27 31^ and relationships with older men, the acute need for girls to engage in transactional sex for resources during the COVID-19 pandemic, along with relationship insecurity, may have widened this power imbalance, leaving girls even more vulnerable to sexual and reproductive harms.

Currently, there are programmes to address gender imbalance within Kenya, such as the Ministry of Health Strategic Plan to acheive gender equality and empowerment for girls and women^32^ which outlines a commitment to making changes and various strategic initiatives to help redress lack of agency. Progress will be measured in part by the indicator: ‘proportion of women aged 15–49 years who make their own informed decisions regarding sexual relations, contraceptive use and reproductive healthcare’. It remains to be seen how successful this is and whether this translates to meaningful empowerment in AGYW agency, particularly in the rural impoverished areas and under future pandemic, or similar situations.

Simultaneously, assessing men’s perceptions of girls’ behaviours during the pandemic enabled us to observe both confirming *and* conflicting perspectives. Inconsistencies within men’s own views included transactional sex,^27 28 33^ when men seemed to believe they were aiding girls, with words such as ‘help’, ‘assist’ and ‘care’ punctuating their narratives. They recognised that girls turned to them from necessity, yet also believed girls were obligated to pay them with their bodies. While sexual reciprocity is socially normative in western Kenya,^33^ narratives implied a conditionality of obligation even during the devastation and uncertainty of the pandemic. Men also portrayed themselves as being intentionally used by schoolgirls and needing condoms to prevent girls transmitting infections, with little consideration of the potential for harm *they* present to girls. These sexual double standards have been described elsewhere showing how it is largely acceptable for men to openly express or act on sexual desire, and that girls and women are often vilified for openly expressing or acting on sexual desires.^34^

A few men described themselves as being helpless to resist girls, suggesting that this was just how they were created, that is, men are compelled to act on what is natural to men. They described situations where a girl’s presence was sufficient to lead to a sexual outcome. We did not assess whether these were mutually agreed acts (whether transactional or not), or whether they had some degree of coercion or violation. To date, there is considerable evidence that violence to girls and women, including that of a sexual coercive nature, are a relatively common phenomena in Kenya.^35^

Few studies exist exploring heterosexual male attitudes and beliefs which drive their sexual behaviours.^36^ Our data suggest that men felt comfortable in our FGDs to voice their ‘helplessness’ in acting on their sexual urges with AGYW, and with little or no disagreement or questioning from their peers about having these sexual encounters with young girls. This indicates that interventions to successfully reduce harms to AGYW and increase their agency will need to engage men and understand their perspectives to meaningfully and effectively shift such gendered stereotypes and norms.^37^

This study had some limitations. Participants were asked to recall phenomena from during the COVID-19-related school closures, which ended approximately 15–18 months prior to these FGDs, and thus sentiments may have changed in retrospect. While findings from FGD narratives between the two gender groups were similar suggesting consistency of views, men’s FGD dynamics may have contributed to more extreme masculine perspectives. Within a male environment, participants may have felt compelled to display a more masculine or male hegemonic attitude than had we interviewed them individually. Using a female moderator may have influenced the discussion, either tempering stated views or possibly inciting them. We suggest that future work uses a mix of qualitative methods including individual interviews to evaluate individual versus group responses. A number of quotes referred to ‘older’ men but we did not receive clarification on what constitutes ‘older’. This may have had some connotations for our study findings, for example, power imbalances, and risks of coercion and abuse. Further exploration of the characteristics of men who wish to ‘help’ or support girls—and who demonstrate a greater understanding of their predicament—could help identify effective messaging and groups to collaborate with in the future as advocates for AGYW.

In conclusion, we highlight the potential long-term risks that COVID-19 restrictions, curfews and school closure posed to girls concerning educational outcomes and SRH harms. Recognising that transactional sex became an even greater necessity due to acute circumstances of poverty, we recommend developing mitigation methods now in preparation for future catastrophic events to prevent girls from once again bearing the brunt of this poverty, with lifelong ramifications. While condom programmes, contraceptive services, STI testing and treatment and access to safe, accessible, affordable abortion services have long been advocated, we can only reiterate their importance. We recommend gaining a deeper understanding of men’s attitudes and behaviours towards AGYW, in order to provide a better platform for collaborating with men in reducing power imbalances and coercion in sexual relationships with AGYW.

## supplementary material

10.1136/bmjph-2024-001214 online supplemental file 1

## Data Availability

Data are available on reasonable request.
