# Supplementary material for: “Whenever I help her, I am also expecting her vagina in return”: a qualitative analysis to explore men’s and adolescent girls’ perceptions of the impact of the COVID-19 pandemic on the sexual behaviour and health of adolescent girls in rural western Kenya
Source: BMJ Public Health. 2024 Nov 14;2(2):e001214. doi: 10.1136/bmjph-2024-001214 (PMC11816869; doi:10.1136/bmjph-2024-001214)
Supplement: online supplemental file 1 [file bmjph-2-2-s001.pdf]

AGYW FOCUS GROUP SCHEDULE
Series 1 – Recall around closure, restrictions and impact on girls

- Overview / warm up

How did you first hear about Covid?
What did you hear about it?
How did you feel when you first heard about it?
What did you think about the restrictions the very first time they were put in place? How did they make you feel?What do you think about Covid now? How does it affect your life at the moment? Do you feel differently about it now compared with when it first started and the schools were closed? Why?

- Changes since school closure

Thinking back to the very first time in 2020 when school had to close from March to September due to Covid, how did you feel about it?
What did you do about your studies during the first school closure? (During March to September 2020) (How did you feel, were you able to study – how, what did you do, what stopped you from studying, looking back is there anything that you would do differently during your time out of school to help with your studies? Is there anything that could have been put in place to have helped you?
When the schools re-opened did you return or not return?

(For those of you who did return….)Why did you return? When you returned to school after the school closure, was anything different in your school life? (school friends/ schoolwork/ homework/ pressures) why, how did this make you feel? What did you do about it?
How did you feel about continuing with schooling during COVID? Was it easier or more difficult? Why?

(For those of you who did not return….) Why did you not return? Was this your own choice? If not, Whose was it? How did you feel about their decision at that time? How did you feel about that person / people making that decision on your behalf? What about now, are you glad that you did not return – why / why not?

(To ask all – those who did return and those who did not) Is there anything that could be put in place to help girls like yourselves to return to school in the future?

- Home life changes

How did you spend your time when you were off school (March 2020) because of COVID closures (why? how did you feel about this?)
How did your being out of school affect your home life? missing school when Covid restrictions first began affect your home life? (Why? how did you feel about this– how did this affect you, what about the rest of the family/household?)
What has changed in your own life since the Covid pandemic began in March 2020? What about living arrangements? Why? How do you feel about it? Working? Why? How do you feel about it? Earning money? Why? How do you feel about it? (For each: Do you think

these changes would have happened if school had stayed open and Covid had not been around?)
<u>How did Covid closures affect your family or those in your households' ability to work?</u> (What about income? How did you manage for money, food etc What were the effects on other families local to you, and the rest of your community?
<u>Other stressors in your home life?</u> What, how, why?
<u>Thinking about your home life, what would have made things better for you, your family or community during this time?</u>

- Sex / relationship life changes

<u>What affect did the Covid pandemic (including restrictions or school closure) have on your relationships?</u> (Family, friends)
<u>What affect did the Covid pandemic (including restrictions or school closure?) have on the relationships with boyfriends or men for girls like you?</u> (fewer or more partners – why? older or younger men – why? were girls like you under more or less pressure to have sex – in what way/s, why, from who – boys, men, family members? Do you think girls like yourselves had more or less sex in order to obtain gifts or money – why was this? How does it make girls feel?...(positive, powerful, sad, scared, normal)…… Do they do this for themselves or for family / others? Can you explain why
<u>Have many of you or your friends from school got married since the covid pandemic began in March 2020?</u> Do you think these marriages would have happened if Covid had not happened? Why / why not? How do you feel about this?
<u>Have many of you or your friends from school had children since the covid pandemic began in March 2020?</u> Do you think many of you or your friends would have had these children if Covid had not happened? Why / Why not? How do you feel about this?
<u>Do you think girls like yourselves who did not return to school have had more or fewer worries about pregnancy during the covid pandemic</u> – in what ways? Why? Do you think more or fewer girls became pregnant during Covid restrictions and closures? Tell us why you think that. What happened when girls like yourselves became pregnant during this time? Do you think many chose to have an abortion? Why would they choose this? How might they go about having one?
<u>Do you think girls like yourselves who did not return to school have had more, or fewer worries about getting a disease like HIV or other sexually transmitted infection during the covid pandemic restrictions / school closure</u> – in what ways? Why?
Do you think girls like yourselves generally worry about getting sexually transmitted infections? Why? Why not? What do girls do if they think they might have an STI? Tell us what happens. If nothing – why not?
<u>What about any other stressors in your social life / relationships?</u> What are these, why do you think they are stressful, how do you deal with them?
<u>Thinking about relationships and sex, what would have made things better for you during the past 2 years of the covid pandemic?</u>

- Changes in health

<u>What effect did the COVID restrictions / school closures have on your physical health</u> – (exercise, food/ diet, ill health……) How, why? how did this make you feel? Where were you

able to seek help? What difference did this make? What else could have helped you at this time? Were there girls like yourselves who needed help with their health during COVID but did not get it? What were some of the health problems they had? [Were they related to pregnancy / STI's / HIV?] What were the reasons they did not get help?
What did girls like yourselves worry about, if anything at all, during the first school closures? (catching covid – (self, family, friends, boyfriends), money, getting behind with study -why?, how did this make you feel? what about any affects on sleep, concentration, appetite, going out socially, going out to do work?
What sort of help would benefit girls who were worried during Covid? – who from? Was this sort of help available? Do you know if girls like yourselves actually sought help during this time to help with this – why/why not? Who from? What happened?
Do you think there were some girls who did not worry about anything to do with Covid / school closures? What sort of girls? why do you think they didn't worry?
Thinking about physical and mental health what would have made things better for you, your family, friends or community during periods of Covid restrictions and school closures?

We know that because school was shut down very quickly some girls left their menstrual cup in their locker so were without it during the school closure. If this happened to any of you, how did you feel about this? what did you do?

- Closure

Thinking about what we have discussed in relation to how covid affected your lives the first time there were restrictions in place, is there anything you feel we have missed out that you want to talk about?
What was the worst thing about the Covid pandemic (including closures/restrictions)?
Thinking ahead, what would be the one most important thing to put in place to help you, if something like Covid was to restrict your lives again?

COMMUNITY MALES FOCUS GROUP SCHEDULE

Series 1 Community Male FGD

- Overview / warm up

How did you first hear about Covid?
What did you hear about it?
How did you feel when you first heard about it?
What did you think about the restrictions? How did they make you feel?

- Function within community generally & during Covid

What role do men like yourselves have in your community? Does it involve leadership (of whom, why? why men like yourselves and not others?) Why do you think this is your role? (Is this role important?) What do you think is important about this role? What do others in your community think about your role (elders, young men & boys, women, young women and girls)
What role did men like yourselves have in your community during Covid & restrictions? do you think your role has changed since the Covid pandemic? In what way? Why? What have you done differently? What do others in your community think about your role during Covid? Did your community look to men like you to lead / help during Covid? (who, why? what did they look to you to do? Why not do this themselves?)

- Role helping vulnerable girls

Were there any ways in which schoolgirls relied on men like yourselves during the school closure & Covid restrictions? What were you been able to do? How did this helped? Could you have done more – how?
Are there any ways in which girls and young women in your community have relied on other groups of men during Covid & restrictions? What groups of men? What did these men do? What is your opinion about this?
We know that some schoolgirls rely on men to provide sponsorship or gifts to help them remain at school, what do you think about this situation? What about if these girls have sex with them in return for their sponsorship or gifts? Why do you think of this? What are these girls like? What is your opinion of them and what they do?
What about girls who are not in school who rely on men to provide them with money or gifts - what do you think about this situation? What about if these girls have sex with them in return for their money or gifts? Why do you think of this? What are these girls like? What is your opinion of them and what they do? Are they different to schoolgirls? In what way? Why? Do men treat them differently to the way they treat schoolgirls? In what way? Why? Do you think men prefer to 'support' schoolgirls or those who have dropped out of school? Why? Are these relationships different? In what way? Why?
The sponsors or those men who give money / gifts to girls, what are they like? What is your opinion of them and what they do? Does it help girls/families, why / not? When and what makes these men stop sponsoring or giving money / gifts to girls? (Pregnancy / school drop-out / earning)
Do you think this type of situation where men give gifts / money or sponsorship to girls should be changed? why? what could be done? Is there anything that men like yourselves could do?
Do you think this situation in which girls rely on men for sponsorship, money or gifts has changed during Covid? In what way? Why? Does it help girls / families, why / not? Are there

groups of men who have particularly been involved in sponsorship / gift giving during these times, what groups of men?

- Role model to students

Do you think men like yourselves can act as role models to school students? In what way? why, different for boys versus girls, why, different for younger girls to older girls, how, why
Do you think this role changed during Covid? – how, why, different for boys versus girls, why, different for younger girls to older girls, how, why
Thinking back, what could / would you have done differently to help students / girls in particular when the schools first closed down and Covid restrictions were first put into place? Why, what would this have achieved? What about now?
